# Primary synovial chondromatosis of the hip joint in children

**DOI:** 10.11604/pamj.2019.33.41.17957

**Published:** 2019-05-21

**Authors:** Zied Jlalia, Dhia Kaffel

**Affiliations:** 1 Pediatric Orthopedics Department, Kassab Institute of Orthopedic Surgery, Ksar Said, Tunisia; 2 Rheumatology Department, Kassab Institute of Orthopedic Surgery, Ksar Said, Tunisia

**Keywords:** Synovial chondromatosis, hip, children

## Image in medicine

A sixteen-year-old patient consulted for right hip pain, with limited mobility and no fever. Standard radiograph of the hip shows widening of the joint space with organized calcifications of the roof of the acetabulum (A). CT scan showed many intra-acetabular osteochondroma (B). The hip was approached anteriorly, without dislocation of the femoral head. Multiple free chondromes and some adherents to the acetabulum were found (C). The diagnosis was confirmed by anatomopathological study. The follow-up was good with recovery of mobility. Primary synovial chondromatosis is a benign synovial dystrophy, characterized by the formation of cartilaginous nodules (chondromas) that may ossify secondarily in a joint or synovial sheath. Its evolution is slow and asymptomatic at its beginning. It is a cause of lameness in children and often poses a problem of differential diagnosis with rheumatic arthritis.

**Figure 1:**
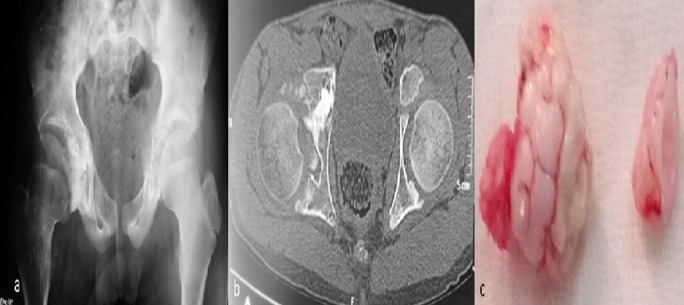
(A) pelvic X-ray, widening of the hip joint space with organized calcifications of the roof of the acetabulum; (B) CT scan showed many intra-acetabular osteochondroma; (C) macroscopic aspect of intraarticular chondroma

